# Phenolics Extracted from *Jasminum sambac* Mitigates Diabetic Cardiomyopathy by Modulating Oxidative Stress, Apoptotic Mediators and the Nfr-2/HO-1 Pathway in Alloxan-Induced Diabetic Rats

**DOI:** 10.3390/molecules28145453

**Published:** 2023-07-17

**Authors:** Urooj Umar, Sibtain Ahmed, Asra Iftikhar, Maryam Iftikhar, Wafa Majeed, Atika Liaqat, Sana Shahzad, Mateen Abbas, Tahir Mehmood, Farooq Anwar

**Affiliations:** 1 Department of Pharmacy, Faculty of Pharmaceutical Sciences, University of Faisalabad, Faisalabad 38000, Pakistan; uroojumar2661@gmail.com (U.U.); asra.iftikhar@tuf.edu.pk (A.I.); atika.liaqat@tuf.edu.pk (A.L.); sshahzad@tuf.edu.pk (S.S.); 2 Department of Biochemistry, Bahauddin Zakariya University, Multan 60800, Pakistan; 3 Institute of Home & Food Sciences, Government College University Faisalabad, Faisalabad 38000, Pakistan; maryamiftikhar72@yahoo.com; 4 Department of Pharmacy, University of Agriculture, Faisalabad 38000, Pakistan; wafamajeed@hotmail.com; 5 Quality Operations Laboratory, Institute of Microbiology, University of Veterinary and Animal Sciences, Lahore 54000, Pakistan; mateen.abbas@uvas.edu.pk; 6 Centre for Applied Molecular Biology (CAMB), University of the Punjab, Lahore 53700, Pakistan; 7 Department of Food Science, Faculty of Food Science and Technology, Universiti Putra Malaysia, Serdang 43400, Malaysia; fqanwar@yahoo.com; 8 Institute of Chemistry, University of Sargodha, Sargodha 40100, Pakistan

**Keywords:** *Jasminum sambac*, alloxan monohydrate, cardiomyopathy, oxidative stress, cardiac function markers

## Abstract

Diabetes mellitus is a chronic metabolic disorder defined as hyperglycemia and pancreatic β-cell deterioration, leading to other complications such as cardiomyopathy. The current study assessed the therapeutic effects of phenolic acids extracted from *Jasminum sambac* phenols of leaves (JSP) against diabetes-induced cardiomyopathy in rats. The rats were divided into four groups, with each group consisting of 20 rats. The rats were given intraperitoneal injections of alloxan monohydrate (150 mg/kg) to induce diabetes. The diabetes-induced groups (III and IV) received treatment for six weeks that included 250 and 500 mg/kg of JSP extract, respectively. In the treated rats, the results demonstrated that JSP extract restored fasting glucose, serum glucose, and hyperlipidemia. Alloxan induced cardiomyopathy, promoted oxidative stress, and altered cardiac function biomarkers, including cardiac troponin I, proBNP, CK-MB, LDH, and IMA. The JSP extract-treated rats showed improved cardiac function indicators, apoptosis, and oxidative stress. In diabetic rats, the mRNA expression of caspase-3, BAX, and Bcl-2 was significantly higher, while Bcl-2, Nrf-2, and HO-,1 was significantly lower. In the treated groups, the expression levels of the BAX, Nrf-2, HO-1, Caspase-3, and Bcl-2 genes were dramatically returned to normal level. According to our findings, the JSP extract prevented cardiomyopathy and heart failure in the hyperglycemic rats by improving cardiac biomarkers and lowering the levels of hyperlipidemia, oxidative stress, apoptosis, hyperglycemia, and hyperlipidemia.

## 1. Introduction

Diabetes mellitus (DM) is a persistent metabolic disorder that is delineated by hyperglycemia and pancreatic β-cell dysfunction, due to which β-cells cannot produce sufficient insulin, ultimately leading to impaired insulin secretion and inappropriate utilization of carbohydrate metabolism disorder in the body [1]. Recent research has reported that oxidative stress is a prime cause of diabetes and its complications. Due to oxidative stress, the number of free radicals increases in the body, eventually resulting in cell death. All these complications lead to various types of abnormalities, including cardiomyopathy, hepatopathy, nephropathy, neuropathy, and retinopathy [2].

Diabetic cardiomyopathy is described as a disease in which irregular myocardial structure and function occurs in diabetic patients, while other risk factors such as hypertension, significant valvular disease, and coronary artery disease are absent. If systolic dysfunction happens due to diabetes, then there is an imperilment for idiopathic cardiomyopathy (ICM), a condition in which systolic function decreases with an enlarged left or right ventricle. The exact cause remains unknown initially in almost 50% of patients; this condition is known as idiopathic dilated cardiomyopathy (DCM). The pathology provides no indication; the heart becomes dilated and the size of the heart also increases, but the thickness of the ventricular walls remains the same [3].

The use of plants and herbal medications is a common practice for the treatment of several ailments such as diabetes mellitus and its complications [4]. *Jasminum sambac* is an ornamental plant that is cultivated all over Asia and it belongs to the *Oleaceae* family. The existence of glycosides, proteins, and coumarins; flavonoids such as rutin, quercetin, kaempferol, and apigenin; phenolics such as caffeic acid, gallic acid, and chlorogenic acid; ascorbic acid, resin, terpenes, saponins, steroids, salicylic acid, and essential oils was confirmed by phytochemical analysis of *Jasminum sambac*. The extract obtained from *Jasminum sambac* has therapeutic activities such as antimicrobial, anti-oxidant, and anti-inflammatory properties. This plant has also been proven to be curative and efficacious in the treatment of several infectious diseases because it contains phenols, cardiac glycosides, carbohydrates, alkaloids, terpenes, and some other constituents [5]. 

The extract from the leaves of *Jasminum sambac* produces a lowered plasma glucose level, serum urea, and improved hyperlipidemia in hyperglycemic rats [6]. The pharmacological basis for the therapeutic uses of *Jasminum sambac* in relation to cardiovascular diseases was investigated in a research study. According to the study, *Jasminum sambac* crude leaf extract demonstrated ex vivo vasorelaxant effects in an endothelium-intact aorta ring preparation, and hypotensive effects were also noted with the aid of the Power Lab. During an ex vivo study, it was discovered that the *Jasminum sambac* leaf extract caused a vasorelaxant/hypotensive action via activating muscarinic receptors and/or producing the local vasodilator nitric oxide [7]. Keeping in view the various therapeutic roles of *Jasminum sambac*, the current experimental study aimed to evaluate the pharmacological effect of phenols extracted from *Jasminum sambac* leaves on diabetes-induced cardiomyopathy in alloxanized hyperglycemic rats.

## 2. Results

### 2.1. Extraction and Phytochemical Analysis of Phenols

Through a maceration procedure, phenols were extracted from *Jasminum sambac* (leaves) dry powder using solvents, including petroleum ether and diethyl ether. By the end of the extraction process, the highest possible quantity of phenols was obtained. Qualitative phytochemical tests were executed on JSP extract, which disseminated the presence of phenols and flavonoids. Quantitative analysis showed a significant amount of phenolic and flavonoid in the JSP extract. The total phenolic and flavonoid contents identified are shown in Table 1, where ad HPLC was used to calculate the amount of various phenolic compounds in the extract. The phenolic compounds found in the JSP extract are shown in Table 2. The findings revealed a sufficiently high (*p* < 0.05) rosmarinic acid content, followed by *p*-coumaric acid levels in the JSP extract.

### 2.2. In Vitro Antioxidant Assay

According to the TAC results, the JSP extract stood out for activity corresponding to 141.24 ± 15.05 mg of ascorbic acid equivalent per gram. An in vitro anti-oxidant assay was carried out with DPPH via the free radical scavenging method. The JSP extract indicated a good dose-dependent antioxidant potential. The DPPH assay was used to anticipate the antioxidant potential by the procedure in which antioxidants inhibited lipid oxidation, resulting in DPPH radical scavenging. The percentage inhibition of DPPH by the JSP extract was 39.56%. The corresponding antioxidant potency of the JSP extract to neutralize the radical ABTS+ was equated with the standard Trolox. The extract showed good scavenging potential of 364.53 ± 7.43 mol Trolox/gram.

### 2.3. Effects of the JSP Extract on the Body Weight, Water, and Feed Intake of the Rats

Body weight, feed, water intake, and fasting blood glucose levels are essential components of health. Therefore, they were assessed at 0, 1, 2, 3, 4, 5, and 6 weeks of the experiment. After induction of diabetes mellitus, the average body weight and feed intake were effectively reduced (*p* < 0.05). Water intake was effectively increased (*p* < 0.05) in the diabetic groups, except for the normal control group. The JSP extract at a dose rate of 250 mg/kg positively impacted the body weight and feed of the diabetic rats in groups III and IV. However, a 500 mg/kg dose produced highly significant results (*p* < 0.01) by prominently increasing the weight and feed intake close to normal. The hyperglycemic rats also showed a remarkable (*p* < 0.01) decline in water intake at a higher dose compared to a low dose of the JSP extract, as shown in Table 3.

### 2.4. Biochemical Analysis

#### 2.4.1. Effects of the JSP Extract on the Fasting Blood Glucose Level in the Rats

**Figure 1:**
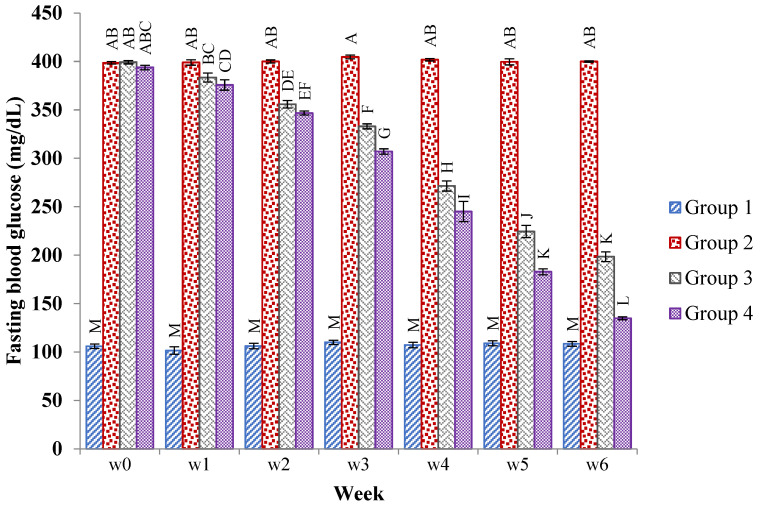
Mean ± SE of the fasting blood glucose levels (mg/dL) of the normal, diabetic, and 250 mg/kg and 500 mg/kg of JSP extract-treated groups of rats at 0, 1, 2, 3, 4, 5, and 6 weeks. Different caps letter in the same bar show significant (*p* < 0.01) differences of means in diabetic, control and treated groups within a bar.

Throughout the study period, the fasting blood glucose level of all groups of rats was checked once a week. Except for the normal control group, the glucose level in the hyperglycemic rats increased effectively (*p* < 0.05) after diabetes induction. However, from the first to the sixth week of the experiment, administration of the JSP extract at 250 and 500 mg/kg, respectively, resulted in lower fasting blood glucose levels in the III and IV groups. The results are shown in Figure 1. 

#### 2.4.2. Effects of the JSP Extract on the Serum Glucose, Serum Insulin, and Glycosylated Hemoglobin Level of the Rats

The serum glucose, glycosylated hemoglobin, and insulin levels were monitored on the first, third, and sixth weeks of the experiment. Except for the normal group, the serum glucose and glycosylated hemoglobin levels were effectively raised (*p* < 0.05), while the insulin level was consequentially reduced (*p* < 0.05) in the diabetic groups. The 250 mg/kg JSP extract gradually lowered (*p* < 0.05) the level of serum glucose, glycosylated hemoglobin and notably (*p* < 0.05) improved the serum insulin concentration in the third group of rats, particularly at the end of research study, in contrast to the diabetic control group depending on the time duration. However, the 500 mg/kg of JSP extract-treated group showed highly remarkable improvement (*p* < 0.01) in the serum glucose and insulin levels (Table 4).

#### 2.4.3. Effects of the JSP Extract on Serum Lipid Profile and Liver Function Enzymes

Except the normal control group, the mean serum cholesterol, serum triglyceride, and LDL levels were significantly enhanced (*p* < 0.05) in the diabetic groups after induction of diabetes, while the level of serum HDL was effectively lowered (*p* < 0.05) in all of the diabetic groups. The results showed that the JSP extract in group III at a dose of 250 mg/kg and in group IV at 500 mg/kg successfully reduced the serum levels of cholesterol, serum triglycerides, and LDL while remarkably raised the serum HDL level in a time- and dose-dependent manner in comparison to the diabetic group. However, 500 mg/kg was the most effective dose for improving the lipid profile in the diseased rats compared to 250 mg/kg.

Except for the normal control group, the mean serum ALT and AST levels were effectively increased (*p* < 0.05) after diabetes induction with alloxan monohydrate. The findings revealed that the JSP extract at both doses of extract produced (*p* < 0.05) a considerable fall in the level of ALT in the treated groups. However, the lower dose produced non-significant changes in the serum AST level compared to the diabetic group. However, the use of a chronic dose of extract efficiently reduced (*p* < 0.05) the serum level of AST in group IV compared to the positive control group (Table 5).

#### 2.4.4. Effects of JSP on Cardiac Oxidative Stress and Antioxidant Enzyme Level 

Cardiomyopathy and diabetic cardio toxicity are linked to oxidative stress. MDA is considered an indicator of oxidative stress. Compared to the normal control group, the MDA level was considerably (*p* < 0.05) elevated in the cardiac tissues of the hyperglycemic rats. However, its level was markedly decreased (*p* < 0.05) by persistent medication with the JSP extract (250 and 500 mg/kg). Antioxidant stress enzymes are essential in preventing oxidative stress and damage to cardiac tissues. The levels of CAT, SOD, and GPx were remarkably lowered (*p* < 0.05) in the myocardial tissues of the alloxanized hyperglycemic rats in contrast to the control rats, indicating a worsening of the antioxidant defense barrier. The lowered levels of antioxidants and CAT were considerably restored (*p* < 0.05) by the use of 250 mg/kg of the JSP extract. However, a 500 mg/kg dose showed highly significant (*p* < 0.01) results by strongly reversing the levels of GPx and catalase to nearly normal compared to the diabetic group (Table 6).

#### 2.4.5. Effects of the JSP Extract on Cardiac Function Biomarkers

**Figure 2:**
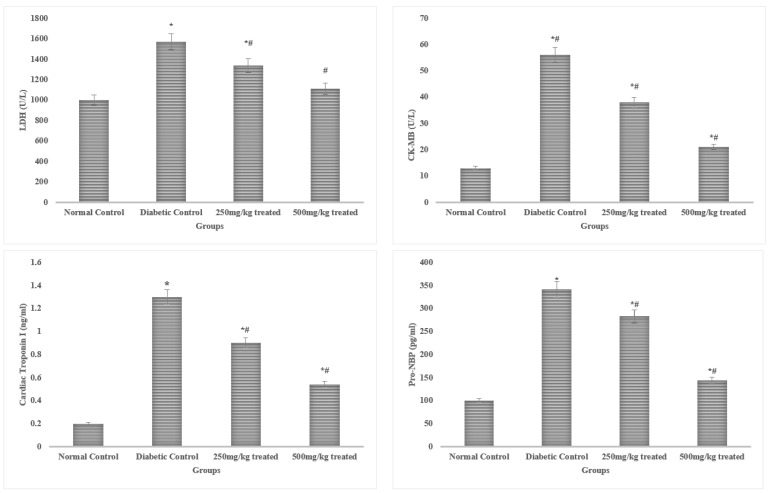

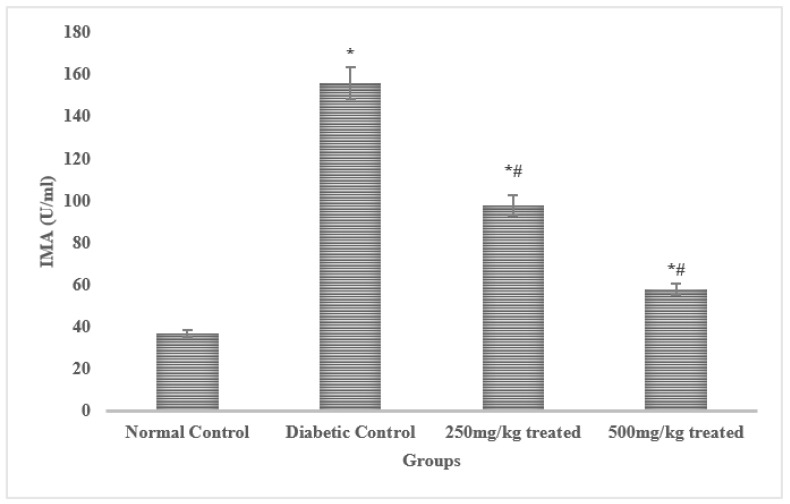
**Effects of the JSP extract on cardiac biomarkers**. * Significant (*p* < 0.05) difference among the normal control and other groups. ^#^ Significant (*p* < 0.05) or *^,#^ highly significant (*p* < 0.01) difference among the diabetic, control and treated groups.

Serum levels of LDH, troponin, and CK-MB are frequently utilized to detect cardiac myopathy, in addition to cardiac dysfunction. The rats exposed to alloxan had considerably higher (*p* < 0.05) serum CK-MB, troponin I, and LDH levels than the non-stimulated control rats. The elevated levels of the aforementioned indicators were remarkably (*p* < 0.05) controlled by the JSP extract therapy. However, a higher dose (500 mg/kg) produced more pronounced (*p* < 0.01) results than a lower dose, resulting in recovery of cardiac function and decreased cardiac myopathy. The progenitor of brain natriuretic peptide (pro-BNP) has been found to be increased in left ventricular deterioration. This peptide’s high production has been linked to coronary artery disease. Serum natriuretic peptide and IMA levels were effectively elevated (*p* < 0.05) compared to the control group in the rats after diabetes induction. The results indicate that a higher dose of the JSP extract produced highly significant effects (*p* < 0.01) in the treated group by efficiently dropping the serum levels of IMA and pro-BNP compared to the diabetic control group. The low dose (250 mg/kg) of the JSP extract also reduced (*p* < 0.05) the serum IMA and pro-BNP levels compared to the diabetic control group, but the effect was low compared to the 500 mg/kg dose (Figure 2).

#### 2.4.6. Histopathological Examination

##### Pancreas

**Figure 3:**
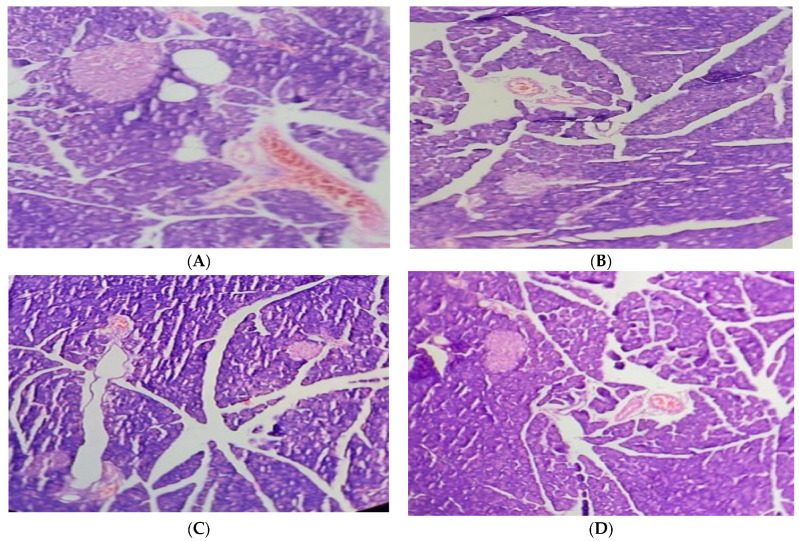
(**A**) Pancreas of the normal control group with normal histological islets of Langerhans, including active nuclei. (**B**) Pancreas of the diabetic control group with inflammatory and necrotic alteration in β-cells, cellular infiltration, vacuolization, congestion, and atrophy. (**C**) Pancreas of the 250 mg/kg-treated group with improvement in cellular damage and congestion. (**D**) Pancreas of the 500 mg/kg-treated group with an increased volume of islet cells and regeneration of β-cells.

The pancreas of the normal control group displayed typical islets of Langerhans histological characteristics, such as effective nuclei and copious cytoplasm. Compared to the healthy control group, the pancreatic tissue of the alloxan-induced diabetic rats exhibited deterioration, atrophy, inflammatory cellular infiltration, vacuolization, substantial necrotic alterations, congestion, and regression in cell size. In the treatment group of hyperglycemic rats, administering 250 mg/kg of the JSP extract resulted in a substantial reduction (*p* < 0.05) in cellular destruction, as shown by partial repair of islets and cells. The diabetic group that received 500 mg/kg of the JSP extract responded by producing more (*p* < 0.05) islet cells overall and fewer necrotic cells, which suggests regeneration. According to the research, islets of Langerhans in the diseased rats administered 500 mg/kg of the phenolic extract displayed a healthy architecture with active cells and no necrotic alterations (Figure 3).

##### Heart

**Figure 4:**
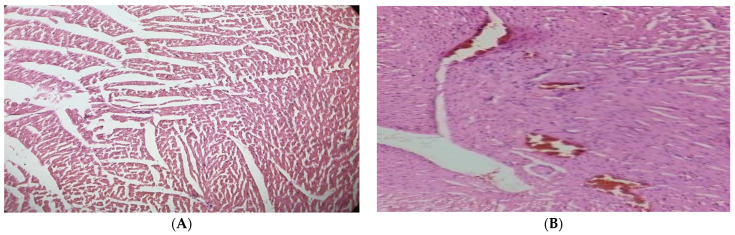

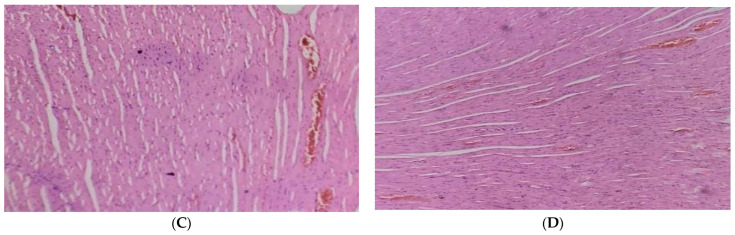
(**A**) Cardiac tissue of the normal control group with normal histology of cardiac myofibril. (**B**) Diabetic cardiac tissue showed fibrosis and disrupted and degenerating muscle fibers with extravagated blood vessels. (**C**) Cardiac tissue of the 250 mg/kg-treated group with partial improvement of disrupted elastic fibers and enlarged myofibrils. (**D**) Cardiac tissue of the 500 mg/kg-treated group showed reversal of cardiac injury with a lesser degree of myocardial damage and mild edema.

According to the findings, contrary to the normal architecture seen in the hearts of the normal control group, histological analysis of the diabetic cardiac tissues revealed fibrosis, degenerating muscle fibers, and disrupted muscle fibers with extravagated blood. In the diabetic rats given 250 mg/kg of the JSP extract, small patches of widely separated myofibrils and virtually normal myocytes were seen. Although, the diabetic group treated with 500 mg/kg showed specific improvements (*p* < 0.05) near normal myocytes, a lesser degree of myocardial damage, and a significant reduction in myocardial edema (Figure 4).

#### 2.4.7. Gene Expression Analysis

**Figure 5:**
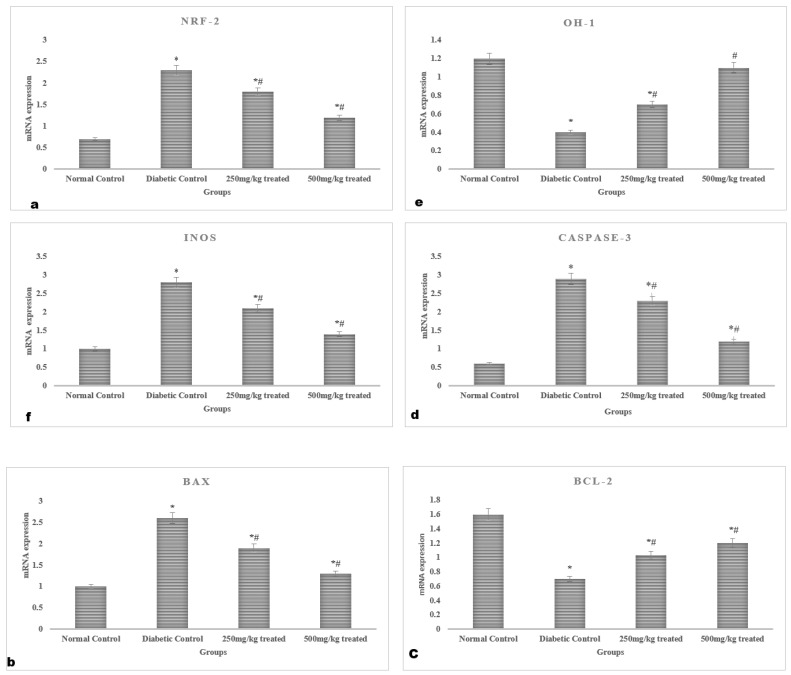
Myocardial gene expression profile of the rats: mRNA expression of (**a**) Nrf-2, (**b**) Bax, (**c**) Bcl-2, (**d**) Caspase-3, (**e**) HO-1, and (**f**) iNOS. * Significant (*p* < 0.05) difference among the normal control and other groups. ^#^ Significant (*p* < 0.05) difference among the diabetic, control and treated groups.

In the hyperglycemic rats, the mRNA expression of Nrf-2, HO-1, and iNOS in the myocardium was downregulated (*p* < 0.05). However, the myocardium’s mRNA expression of Nrf-2 and HO-1 were both increased by JSP treatment, showing that the antioxidant defense had been restored. The use of JSP extract no antioxidant declined the iNOS mRNA expression. Furthermore, treatment with a high dose (500 mg/kg) produced more pronounced results compared to a lower dose (250 mg/kg). The expression of the anti-apoptotic protein Bc1-2 was significantly lowered (*p* < 0.05) in the myocardia of the hyperglycemic rats in contrast to the normal rats. However, the mRNA expression of apoptotic genes caspase-3 and Bax was greatly enhanced (*p* < 0.05). In comparison, in the diabetic rats, the chronic administration of graded doses of the JSP extract considerably lowered (*p* < 0.05) the caspase-3 and Bax levels and raised the Bcl-2 mRNA expression (Figure 5).

## 3. Discussion

Medicinal plants have been used for years to medicate many health-related complaints and meet patients’ nutritional requirements. Herbal medicines have also been used for decades for the treatment of diabetes, along with its complications. These drugs contain many phytochemical components, each with a distinct structure, which work together to cure various illnesses [8]. The most important phytochemical group with anti-inflammatory, antistress, antioxidant, and antihyperglycemic characteristics is the phenolic group. In our study, the phenolic concentration in the JSP extract was 129.6 ± 3.0 mg GAE/g. Because of their antioxidant and antihyperglycemic properties, phenolic extracts are accountable for therapeutic efficacy against diabetes and its complications. These phenolic compounds influence the glucose metabolism through various mechanisms, including activation of insulin release from pancreatic cells, insulin receptor activation, glucose uptake in insulin-sensitive cells, increased glucokinase activity, and glucose release control from the liver [9].

In a recent study, alloxan monohydrate was used to produce diabetes, resulting in hyperglycemia [10]. Under diabetic conditions, the body mass of the diabetic rats was considerably decreased because there was a decrease in the level of insulin in the body, and the body’s ability to absorb glucose was diminished [11]. In this context, the body starts to burn fats, thus resulting in overall weight loss. Oral administration of the JSP extract gradually increased (*p* < 0.05) body weight, as well as lowered the fasting blood glucose level depending on the time and dose of the drug. In a hyperglycemic state, due to the eradication of glucose homeostasis, the levels of Hb1Ac and insulin in the body are disturbed [12]. The results of this study showed that the JSP extract considerably reverted (*p* < 0.05) the serum glucose, HbA1c, and insulin levels in the treated groups. Compared to the alloxan-induced diabetic rats, JSP extract therapy reduced the rise in cardiac parameters, hyperlipidemia, and hyperglycemia, with a comparative reduction in body weight [13,14].

The results revealed that the levels of cardiac troponins I, pro-BNP, and IMB were elevated effectively (*p* < 0.05) in the diabetic rats. An increase in troponin in diabetic individuals with numerous cardiovascular risk factors is linked to chronic coronary artery disease. Troponin might be used as a tracer in high-risk groups [15]. The levels of brain natriuretic peptides (Nt-proBNP and BNP) increase in left ventricular dysfunction. A high production of these peptides has been linked to coronary artery disease [16]. Administration of graded doses of the JSP extract in the third and fourth groups of hyperglycemic rats gradually lowered (*p* < 0.05) the cardiac troponins I, pro-BNP, and IMB levels. These improved cardiac troponins I, pro-BNP, and IMB levels predict the antioxidant properties of the JSP extract. These findings are in agreement with a previous study, which also identified the effects of a *Marigold hydroalcoholic* extract on diabetes-induced cardiotoxicity with a remarkable reduction (*p* < 0.05) in cardiac biomarkers [17]. An upraised serum level of CK-MB is a possible and sensitive sign to determine the likelihood of cardiac problems [18]. The current work also showed that hyperlipidemia, another component attributed to cardiac dysfunction and cell death in diabetes, is related to increased CK-MB and troponin I in the serum of rat models of myocardial degeneration. The link between hyperlipidemia and increased triglyceride and cholesterol deposition produces toxicological effects on the heart [19].

Diabetes mellitus is frequently accompanied by hyperglycemia, insulin resistance, inflammation, and dyslipidemia, all of which promote the production of reactive oxygen species (ROS), which are recognized to be a factor in the development of diabetic ailments such as cardiomyopathy [20]. The buildup of ROS and superoxide produces oxidative stress, which can cause DNA damage, cardiac cell death, myocardial contractility loss, and cardiac fibrosis. Additionally, the buildup of ROS/RNS causes degradation of the antioxidant defense system, which is made up of scavenger antioxidating GPx, SOD, and catalase [21]. Our findings show that the GPx, SOD, and catalase levels were remarkably lower (*p* < 0.05) after the induction of diabetes. In contrast, continuous use of the JSP extract had a dose-dependent ability to restore depleted antioxidant enzymes to their normal levels. Strong markers of antioxidants’ preventive effect include the recovery of antioxidant enzymes (GPx, CAT, and SOD) and the lowering of antioxidants. These findings are consistent with earlier literature [22]. 

Numerous cytoprotective proteins, such as heme oxygenase-1 (HO-1), an enzyme that catalyzes the breakdown of heme into the antioxidant biliverdin, carbon monoxide (an anti-inflammatory and antioxidant), and ferrous iron, may be induced by Nrf2 activation [23]. In response to stress factors like oxidative stress, heavy metals, nitric oxide, hypoxia, and cytokines, HO-1 is highly induced. In the antioxidant reaction, the Nrf2/HO-1 pathway always plays a crucial role [24]. To examine the therapeutic role of the JSP extract against induced oxidative stress, the expression of Nrf2 and HO-1 was observed. In line with prior research [25], JSP extract therapy increased the expression of HO-1 and Nrf2 in the treated groups, encouraging the restoration of antioxidant enzymes (GPx, SOD, and CAT). The NO synthase (NOS), which consists of endothelial NOS, neuronal NOS, and iNOS, is responsible for producing nitric oxide (NO). Depending on the disease risk factors, NO-mediated actions might be advantageous or detrimental [26]. Only specific stresses, such as those present in obesity and diabetes, can induce inducible NOS (iNOS) [27]. It has been previously noticed that the proliferation of cardiac fibroblasts is promoted by heart-specific iNOS overexpression in female rats [28]. One of the primary causes of diabetic myopathy is mitochondrial dysfunction, which is encouraged by oxidative stress and results in the activation of pro-apoptotic proteins Bax and caspase-3 and a decrease in anti-apoptotic proteins. Cell survival or death in response to apoptotic stimuli is determined by the Bax/Bcl-2 ratio. It had been demonstrated that increasing Bax expression and decreasing Bcl-2 both encourage cell death [29]. The ratio of Bax/Bcl-2, considered an index of apoptosis, was higher in the diseased group of rats, and JSP extract treatment significantly prevented (*p* < 0.05) this increases in Bax/Bcl-2 ratio in the treated groups. 

It is widely acknowledged that a significant increase in the levels of circulating cardiac damage indicators like troponin and CK-MB serves as a reliable predictor of increased cardiac problems [30]. The alloxanized hyperglycemic rats in this study showed a considerable increase in heart injury indicators, as seen by the degeneration of myocardial tissue, vacuolation, apoptotic nuclei, and necrosis. The JSP extract significantly regularized (*p* < 0.05) the histoarchitecture of pancreatic tissues, according to histopathological examination. In those rats treated with JSP, a dose-dependent increase in the pancreatic beta cell population and a decrease in necrotic tissue were seen. Apoptosis caused by oxidative stress has been shown to be reduced by the phenols found in traditional plants, which could be the reason for pancreatic B-cell regeneration [31]. Administration of the JSP extract to the diabetic rats reduced heart damage and restored the heart cells and nuclei’s histological organization.

## 4. Methods 

### 4.1. Extraction Procedure of Phenols

The leaves of *Jasminum sambac* were used for this experiment. *Jasminum sambac* leaves were collected from the University of Agriculture, Faisalabad (UAF) botanical garden, and verified by the department of botany. Following verification, the plant material was stored in the UAF, Faisalabad, herbarium with voucher number 21,189. After cleaning with water to remove dirt and other unwanted materials, the gathered plant matter (leaves) was dried in the shade. The dried *Jasminum sambac* leaves were powdered and extraction was carried out with 70% ethanol. To acquire residue, the extract was evaporated until it was completely dry. The acquired residue was then treated with petroleum ether and diethyl ether, forming two layers. The aqueous layer containing phenols was collected and utilized for further research [32].

### 4.2. Qualitative Phytochemical Analysis of Phenols and Flavonoids

For the detection of phenols, a few drops of 10% FeCl_3_ were added to the extract, and the appearance of a green color indicated the presence of phenols. For further confirmation of phenols, the extract was dissolved in 10% lead acetate, and bulky white preparation stated the presence of phenols [33]. To detect flavonoids, a few drops of dilute NaOH were added to 1 mL of extract, and a yellow color indicated the presence of flavonoids [34].

### 4.3. Quantitative Phytochemical Analysis of Phenols and Flavonoids

The entire phenolic content in the extract was achieved using the Folin–Ciocalteu method. The absorbance at 765 nm was monitored using a UV-visible spectrometer to achieve determination. The outcomes were compared using gallic acid as the reference component, and are shown as milligram Gallic acid equivalent per gram of extract (mg GAE/g) [35]. The total flavonoid content was calculated by using the aluminum chloride calorimetric technique. The absorbance was determined at 510 nm by spectrophotometer, and the results were demonstrated as milligram catechin equivalent per gram of the extract (mg CE/g).

### 4.4. Quantification of the Phenols in the Jasminum sambac Extract

High-performance liquid chromatography–mass Spectrometry (HPLC/MS, Agilent Technologies, Santa Clara, CA, USA) was used to analyze the *Jasminum sambac* polyphenol (JSP) extract. A C-18 column (4.6 × 250 mm, as well as 5 µm Agilent, USA) was used to separate the different components of the extract. Glacial acetic acid at a concentration of 2% (*v*/*v*) was used to mobile phase A, while mobile phase B was made up of acetonitrile. The analytical conditions were: 10 B, 10 min; 25% B, 15 min; 50% B, 45 min; 75% B, 65 min; 100% B, 75 min. The column temperature was 28 °C, the injection volume was 20 µL, and the flow rate was 1 mL/min. The absorbance wavelength was 280–310 nm for identifying phenolic content using a UV detector. At least three separate phenolic extractions were carried out and evaluated using HPLC/MS. Phenolic substances were measured by comparing each molecule to the standard [36].

### 4.5. In Vitro Antioxidant Analysis of the JSP Extract

#### 4.5.1. Total Antioxidant Capacity (TAC)

The procedure described by Prieto et al. [37] was used to calculate the total antioxidant capacity of the JSP extract with slight modifications. A 0.5 mL extract was mixed with 3 mL of the reagent mixture (28 mM sodium phosphate, 0.6 M sulfuric acid, and 4 mM ammonium molybdate), incubated at 95 °C for 90 min, and the results were compared to the blank at 695 nm. Ascorbic acid was employed as the calibration standard. The results are presented in terms of milligrams of AA per gram of wet weight.

#### 4.5.2. DPPH Assay

For the 1,1 diphenyl-2-picryal hydrazyl (DPPH) assay of the JSP extract, 900 µL of DPPH was added under dark light with 0.1 mL of JSP at various concentrations. The reaction mixtures were kept at room temperature. The prepared sample was incubated for 40 min, and the absorbance of the sample was recorded at 517 nm. The assays were all run in triplicate, and the ascorbic acid equivalent was used to calculate the parentage inhibition [38]. 

By using the following equation, the radical scavenging activity of the phenolic extract was determined:

Inhibition % = 1−(A standard−A blank÷A control) × 100

#### 4.5.3. ABTS•+ Radical Scavenging Assay

The 2,2′-azino-bis(3-ethylbenzothiazoline-6-sulfonic acid) (ABTS•^+^) radical cation-based assay was employed to assess the antioxidant capacity of the JSP extract [39]. The reaction between 7 mM ABTS in water and 2.45 mM potassium persulfate (1:1) created the ABTS•+ cation radical, which was then kept at room temperature for 12–16 h in the dark. The ABTS•+ solution was diluted with methanol to produce an absorbance of 0.700 at 734 nm. Absorbance was measured 30 min after mixing 4 mL of the diluted ABTS^·+^ solution and 5 μL of the extract. Trolox was used as the standard substance and the results are presented in terms of mole Trolox/gram.

### 4.6. Experiment Animals 

Young Wistar albino rats in good health, weighing 180–200 g, were acquired from the animal house of the Department of Pharmacy, University of Faisalabad, Pakistan. The rats were retained in the animal room situated in the Department of Pharmacy. The animals were housed for a two-week acclimation period before the experiment. They were housed in four cages with a consistent 12 h cycle of light and darkness and they received a normal basal diet and water ad libitum. The rats were fed a typical standard diet in the form of 50 g pellets composed of maize (48%), soyabean (20%), corn bran (6.8%), palm kernel cake (5%), fish and bone meal (4% and 3%, respectively), ground nut (12%), and salt (0.25%), with crude proteins (23%) and crude fiber (3%) and balanced with vitamins and minerals. The University of Faisalabad’s institutional review board (IRB) provided ethical approval (TUF/IRB/180/23) to follow the experimental protocols. The research study was conducted in accordance the Guide for the Care and Use of Laboratory Animals by National Research Council (NRC). 

### 4.7. Acute Oral Toxicity Test

The selection of doses of the JSP extract was based on the acute oral toxicity test. The method of OECD Guidelines [40] was used for determining acute oral toxicity in the rats. The rats were divided into five groups, with three animals in each, along with one control group. The animals were kept on water only for a whole night before receiving doses of 1000, 1500, 2000, and 2500 mg/kg of the JSP extract in groups II, III, IV, and V, respectively, for the determination of safe doses. The rats were observed for 14 days after dosage for lethargy, jerkiness, and mortality. The safe dose was determined to be between 1/5th and 1/10th of LD_50_. In the acute oral toxicity dose test, no death or morbidity was seen at any dose. Therefore, the LD_50_ may be greater than 2500 mg/kg. As a result, the maximal safe dosages of 250 and 500 mg/kg were chosen for pharmacological tests [41].

### 4.8. Experimental Design

Following the adaption phase, the animals were split into four groups, each with 20 rats. According to previous studies conducted regarding diabetes induction in rats through alloxan monohydrate, a single shot of alloxan monohydrate (150 mg/kg) mixed with 0.9% normal saline was provided intraperitoneally for diabetes induction in the overnight-fasted rats, except in the normal group. The most important problem associated with use of alloxan is triphasic blood glucose response in animals with initial hyperglycemia due to the apoptosis of beta cells, profound hypoglycemia for approximately 6–12 h, and persistent hyperglycemia after 24 h. Therefore, the rats were given a 5% glucose solution orally during the first 24 h to prevent any negative effects of hypoglycemia. Animals can exhibit signs of illness such as reduced mobility or lethargy due to triphasic response. Thereby, the physical activity of the diseased rats was also continuously monitored for 48 h in order to reduce the chances of morbidity or mortality. 

The blood sugar of all groups of rats was measured using a glucometer via the tail vein one week after the injection. In the study, the diabetic rats were determined to have blood glucose levels higher than 300 mg/dL. During this time period, the animals were given access to water and their regular meal. After confirmation of disease induction (seven days after alloxan induction), treatment of the diseased rats with the test formulation was started by dividing them in groups, and the blood glucose level of all groups was monitored by checking the glucose level of the rats in order to observe the glycemic status and effect of the JSP extract in the treated groups up until six weeks of study. The rats were organized into four groups as follows:

- **Group I:** Untreated normal control group on a routine diet;
- **Group II:** Untreated diabetic (positive control) given alloxan (150 mg/kg);
- **Group III:** Diabetic group treated with the JSP extract (250 mg/kg) for six weeks;
- **Group IV:** Diabetic group treated with the JSP extract (500 mg/kg) for six weeks.

### 4.9. Physical Parameters

#### 4.9.1. Body Weight

Each rat’s body weight was measured weekly until six weeks into the experimental study. 

#### 4.9.2. Feed and Water Intake

Each rat’s feed and water consumption were measured every day up until six weeks of the experimental study. From the data, the average feed and water intake of each group was calculated.

### 4.10. Blood Sampling

After the six-week period of observation, the overnight-fasted rats were sedated with 3% intraperitoneal sodium pentobarbital and then slaughtered. Blood samples were taken from every rat. The gathered blood samples were centrifuged at 3000 rpm at 4 °C for 15 min. The plasma and serum were separated and kept at −20 °C for hormonal and biochemical testing. The heart and pancreas were kept in a 10% buffer formalin solution for histopathological assessment.

### 4.11. Preparation of Tissue Homogenate

The tissues of the rats from each group were kept in 10% (*w*/*v*) buffer (50 mM Tris-HCL, 1.15% KCL, and pH 7.4) and centrifuged at 9000 rpm at 4 °C for 3 min. The obtained tissue homogenate was used for the biochemical analysis. According to the procedure [42], the obtained homogenate’s protein concentration was examined. 

### 4.12. Biochemical Analysis

The fasting blood glucose level was measured by glucometer weekly through the tail prick method up to six weeks of the experiment. The serum glucose and insulin concentrations were estimated by a rat glucose assay kit (81693, crystal chem, Elk Grove Village, IL, USA) and an insulin ELIZA kit (ELR-insulin, RayBio^®^, Peachtree Corners, GA, USA). Glycosylated hemoglobin was estimated using a rat hemoglobin Hb1Ac assay kit (MBS2033689, My BioSure, Grass Valley, CA, USA). The serum triglyceride, high-density lipid (HDL), low-density lipid (LDLs), and cholesterol levels were calculated using commercially available kits (ab65359, ab65336, and ab65390, respectively; Abcam, Cambridge, UK). The serum level of liver function enzymes, i.e., alanine aminotransaminase (ALT) and aspartate aminotransferase (AST), were determined through the kit assay method (ab105134/k752-100 and ab105135, respectively; Abcam, Cambridge, UK).

### 4.13. Oxidative Stress and Antioxidant Enzyme Measurements

A thiobarbituric acid reactive substances (TBARS) assay kit was used to measure lipid peroxidation in the form of malondialdehyde (MDA) using tissue homogenate, according to the method of [43]. The resultant TBARS absorbance was calibrated at 532 nm using a spectrophotometer, and the outcomes were presented as nmol MDA/mg protein. Assays for antioxidant enzymes such as glutathione peroxidase (GPx), superoxide dismutase (SOD), and catalase (CAT) were performed on the tissue homogenate via a catalase activity assay kit (ab83464, Abcam, UK), a SOD assay kit (706002, Cayman chemical, Ann Arbor, MI, USA), and a glutathione peroxidase assay kit (ab102530, Abcam, UK), respectively. 

### 4.14. Determination of Cardiac Biomarkers

The cardiac troponin I level was determined using a commercially available ELISA kit (Cardiac troponin ELISA Kit, ab223860, Abcam, UK). To determine natriuretic peptide (pro-BNP), an ELISA kit (ab263877, Abcam, UK) was used as per the manufacturer’s instructions. The ischemic-modified albumin (IMA) level was analyzed through an IMA ELISA kit (MBS263569) based on IMA’s ability to bind to cobalt. Lactate dehydrogenase (LDH) was investigated through the kit method (MBS269777, MyBioSource, San Diego, CA, USA) and the serum creatinine kinase-MB fraction (CK-MB) level was determined using a CK-MB ELISA kit (CSB-E14403r, CUSABIO, Houston, TX, USA).

### 4.15. Histopathological Examination

The animals were slaughtered at the end of the research session, and organs from the body were separated. The hearts and pancreases of the rats were placed in 10% buffered formalin. The hearts were cut into 3 mm pieces. The organs were sliced perpendicularly, starting from the apex and leading to the base. Sliced heart and specimens from the pancreas were put into paraffin, stained with hematoxylin and eosin, and examined beneath a light microscope [44].

### 4.16. RNA Extraction and Real-Time Quantitative PCR

The TRIzol technique (Thermo Fisher Scientific, Waltham, MA, USA) was used to extract the RNA, which was then modified and measured using the nanodrop technique [45]. A RevertAid cDNA synthetic kit (Thermo Fisher Scientific) was used to reverse transcribe the total extracted mRNA to cDNA, as specified by the manufacturer’s instructions. The maxima SYBR Green/ROX qRT-PCR Master Mix (Thermo Fisher Scientific) performed real-time qPCR (RT-qPCR) on the iQ5 Bio-Rad equipment. Rat primers for gene expression of the Nrf-2, Bcl-2, Bax, Caspase-3, HO-1, and β-Actin genes (Table 7) were used [46] as per the manufacturer’s guidelines.

The PCR conditions were 40 cycles for cDNA denaturation at 95 °C for 15 s, 25 s of annealing at 52 °C, and 20 s of extension at 72 °C. The relative expression of the genes was determined using the 2^−ΔΔCT^ technique. 

### 4.17. Statistical Analysis

The results are presented as the mean and SD. The data were statistically analyzed using Duncan’s multiple range test (DMR) and ANOVAs. *p* < 0.05 was used to determine statistical significance [47].

## 5. Conclusions

The present study revealed that the JSP extract has great potential, with a protective and therapeutic role against diabetic cardiomyopathy. The JSP extract exhibited an excellent ability to decrease cardiac dysfunction through amelioration of oxidative stress, hyperglycemia, hyperlipidemia, and inflammation. Moreover, the JSP extract demonstrated a possible therapeutic approach for managing diabetic cardiomyopathy and cardiac dysfunction, which may be accompanied by the downregulation of cardiac apoptosis-related genes. Thus, these findings support the effectiveness of the JSP extract as a cardioprotective drug and indicate that it may be used as a complementary treatment to treat or prevent diabetic cardiomyopathy and its consequences. However, by conducting systematic research on the JSP extract, such as safety evaluations, molecular mechanism investigations, clinical trials, and dosage formulation studies, it is possible to gain important insights into the potential of various JSP extract formulations. Therefore, there is need to explore the detailed mechanism of the JSP extract with the ultimate goal of transferring these discoveries for use in humans for the management and potential cure of diabetes-associated cardiac abnormalities, which will ultimately benefit millions of people across the world.

## Figures and Tables

**Table 1:** Quantitative determination of the phenol and flavonoid contents of the JSP extract.

Sample	Total Phenolic Content (mg GAE/g)	Total Flavonoid Content (mg CE/g)
JSP extract	129.6 ± 3.0	63.026 ± 3.0

Findings are presented as mean ± SE (*n* = 3).

**Table 2:** The quantity of various phenolics in the JSP extract determined using HPLC.

No. Sample	Name of Phenolics	Quantity (μg/g)
1	Rosmarinic acid	788.7 ± 1.42
2	*p*-coumaric acid	410.2 ± 5.04
3	*p*-hydroxybenzoic acid	188.1 ± 2.36
4	Syringic acids	72.3 ± 3.53
5	Chlorogenic acid	30.7 ± 2.14

Amounts are presented as the mean ± SE (*n* = 3).

**Table 3:** Effect of the JSP extract on body weight, feed, and water intake of the hyperglycemic rats.

Parameters	Normal Control	Diabetic Control	250 mg/kg of JSP Extract	500 mg/kg of JSP Extract
**Body weight (g)**	164.94 ± 1.37 A	104.29 ± 3.65 B *	125.51 ± 2.55 D ^#^	136.31 ± 3.57 C ^##^
**Feed intake (g)**	27.17 ± 0.29 A	20.17 ± 0.57 BC *	22.63 ± 0.48 C ^#^	25.03 ± 0.67 B ^##^
**Water intake (mL)**	45.43 ± 0.59 C	66.17 ± 1.53 A *	57.09 ± 1.29 B ^#^	49.89 ± 1.79 BC ^##^

Results expressed as the mean ± SE; *n* = 20 per group. Significant (* *p* < 0.05) difference between the normal and diabetic groups. ^#^ Significant (*p* < 0.05) or ^##^ highly significant (*p* < 0.01) in same row, while capital letter shows the difference between the diabetic, control and treated groups within a column.

**Table 4:** Effects of the JSP extract on the serum glucose, serum insulin, and glycosylated hemoglobin levels of the hyperglycemic rats.

Parameters	Normal Control	Diabetic Control	250 mg/kg of JSP Extract	500 mg/kg of JSP Extract
**Serum glucose (mL)**	104.60 ± 1.18 D	354.07 ± 9.83 A *	321.53 ± 19.52 B ^#^	283.47 ± 29.47 C ^##^
**Serum insulin (mg/dL)**	12.22 ± 0.28 A	6.49 ± 0.22 D *	8.25 ± 0.50 C ^#^	9.51 ± 0.58 B ^##^
**Glycosylated hemoglobin (mmol/mol)**	5.75 ± 0.13 C	12.82 ± 0.14 A *	11.17 ± 0.48 AB ^#^	10.22 ± 0.70 B ^#^

Results are expressed as the mean ± SE; *n* = 20 per group. Significant (* *p* < 0.05) difference between the normal and diabetic groups. ^#^ Significant (*p* < 0.05) or ^##^ highly significant (*p* < 0.01) in same row, while capital letter shows the difference between the diabetic, control and treated groups within a column.

**Table 5:** Effects of the JSP extract on the serum lipid profile and liver function enzymes of the hyperglycemic rats.

Parameters	Normal Control	Diabetic Control	250 mg/kg of JSP Extract	500 mg/kg of JSP Extract
**Serum cholesterol (mg/dL)**	144.27 ± 2.15 D	252.27 ± 7.92 A *	223.60 ± 4.03 B ^#^	203.40 ± 8.30 C ^##^
**Serum triglycerides (mg/dL)**	99.07 ± 1.70 D	176.73 ± 3.19 A *	149.47 ± 2.64 B ^#^	130.47 ± 6.21 C ^##^
**Serum HDL (mg/dL)**	44.27 ± 1.07 A	30.40 ± 0.68 D *	34.67 ± 1.47 C ^#^	37.80 ± 1.95 B ^##^
**Serum LDL (mg/dL)**	118.13 ± 2.27 D	177.60 ± 2.45 A *	155.87 ± 3.40 B ^#^	146.67 ± 5.05 C ^##^
**Serum ALT (U/L)**	17.80 ± 0.86 C	42.67 ± 1.37 A *	35.67 ± 1.82 B ^#^	34.67 ± 3.14 B ^#^
**Serum AST (U/L)**	21.15 ± 0.63 C	38.40 ± 3.45 A *	36.53 ± 2.31 A ^NS^	32.60 ± 2.59 B ^#^

Results are expressed as the mean ± SE; *n* = 20 per group. Significant (* *p* < 0.05) difference between the normal and diabetic groups. ^#^ Significant (*p* < 0.05) or ^##^ highly significant (*p* < 0.01) in same row, while capital letter shows the difference between the diabetic, control and treated groups within a column.

**Table 6:** Effects of the JSP extract on the oxidative and antioxidant enzyme levels in the hyperglycemic rats.

Parameters	Normal Control	Diabetic Control	250 mg/kg of JSP Extract	500 mg/kg of JSP Extract
**TBRAS** (μM/mg protein)	4.27 ± 0.15 C	10.54 ± 2.1 A *	6.21 ± 1.03 BC ^#^	7.40 ± 1.40 B ^#^
**GPx** (units/mg protein)	148 ± 4.35 A	115.4 ± 3.6 D *	123.5 ± 9.19 C ^#^	141.34 ± 3.11 B ^##^
**SOD** (units/gram tissue)	9.68 ± 1.05 C	15.40 ± 1.42 A *	12.42 ± 1.47 B ^#^	14.64 ± 1.95 AB ^#^
**Catalase** (μmol H_2_O_2_/min/mg protein)	53 ± 2.03 A	23.5 ± 1.48 C *	34 ± 1.04 BC ^#^	43.5 ± 1.54 B ^##^

Results expressed as the mean ± SE; *n* = 20 per group. Significant (* *p* < 0.05) difference between the normal and diabetic groups. ^#^ Significant (*p* < 0.05) or ^##^ highly significant (*p* < 0.01) in same row, while capital letter shows the difference between the diabetic, control and treated groups within a column.

**Table 7:** Primer sequence of genes.

Names	Forward:	Reverse
Nrf2	5′TTTGTAGATGACCATGAGTCGC-3′	5′TGTCCTGCTGTATGCTGCTT-3′
Bax	5′CCTGAGCTGACCTTGGAGCA-3′	5′GGTGGTTGCCCTTTTCTACT-3′
Bcl-2	5′TGATAACCGGGAGATCGTGA	5′AAAGCACATCCAATAAAAAGC
caspase-3	5′CTCGGTCTGGTACAGATGTCGATG	5′GGTTAACCCGGGTAAGAATGTGCA
Beta-actin	CGAGTACAACCTTCTTGCAGC	TATCGTCATCCATGGCGAACTG
HO-1	5′TCTGCAGGGGAGAATCTTGC	5′TTGGTGACGGAAATGTGCCA
iNOS	5′-CATTCAGATCCCGAAACGTAC-3′	5′-AGCCTCATGGTGAACACGTTCT-3′

## Data Availability

Data collected and analyzed during the current research study are available on reasonable demand.

## Sample Availability

Samples of the compounds JSP are available from the authors.

## References

[1] Xiong W., Xiao M.Y., Zhang M., Chang F. (2016). Efficacy and safety of canagliflozin in patients with type 2 diabetes: A meta-analysis of randomized controlled trials. Medicine.

[2] Harding J.L., Pavkov M.E., Magliano D.J., Shaw J.E., Gregg E.W. (2019). Global trends in diabetes complications: A review of current evidence. Diabetologia.

[3] Murthy V.L., Naya M., Foster C.R., Gaber M., Hainer J., Klein J., Dorbala S., Blankstein R., Di Carli M.F. (2012). Association between coronary vascular dysfunction and cardiac mortality in patients with and without diabetes mellitus. Circulation.

[4] Chen J., Mangelinckx S., Adams A., Wang Z.-T., Li W.-L., De Kimpe N. (2015). Natural Flavonoids as Potential Herbal Medication for the Treatment of Diabetes Mellitus and its Complications. Nat. Prod. Commun..

[5] Qi X., Chen S., Wang Y., Feng J., Wang H., Deng Y. (2020). Complete chloroplast genome of *Jasminum sambac* L. (*Oleaceae*). Braz. J. Bot..

[6] Jia G., Whaley-Connell A., Sowers J.R. (2017). Diabetic cardiomyopathy: A hyperglycaemia- and insulin-resistance-induced heart disease. Diabetologia.

[7] Khan I.A., Hussain M., Munawar S.H., Iqbal M.O., Arshad S., Manzoor A., Shah M.A., Abbas K., Shakeel W., Syed S.K. (2021). *Jasminum sambac*: A Potential Candidate for Drug Development to Cure Cardiovascular Ailments. Molecules.

[8] Matowa P.R., Gundidza M., Gwanzura L., Nhachi C.F.B. (2020). A survey of ethnomedicinal plants used to treat cancer by traditional medicine practitioners in Zimbabwe. BMC Complement. Med. Ther..

[9] Kang G.G., Francis N., Hill R., Waters D., Blanchard C., Santhakumar A.B. (2019). Dietary Polyphenols and Gene Expression in Molecular Pathways Associated with Type 2 Diabetes Mellitus: A Review. Int. J. Mol. Sci..

[10] Xu X., Shan B., Liao C.-H., Xie J.-H., Wen P.-W., Shi J.-Y. (2015). Anti-diabetic properties of *Momordica charantia* L. polysaccharide in alloxan-induced diabetic mice. Int. J. Biol. Macromol..

[11] Yang C.S., Zhang J., Zhang L., Huang J., Wang Y. (2015). Mechanisms of body weight reduction and metabolic syndrome alleviation by tea. Mol. Nutr. Food Res..

[12] Rollins K.E., Varadhan K.K., Dhatariya K., Lobo D.N. (2016). Systematic review of the impact of HbA1c on outcomes following surgery in patients with diabetes mellitus. Clin. Nutr..

[13] Jafarnejad S., Keshavarz S.A., Mahbubi S., Saremi S., Arab A., Abbasi S., Djafarian K. (2017). Effect of ginger (*Zingiber officinale*) on blood glucose and lipid concentrations in diabetic and hyperlipidemic subjects: A meta-analysis of randomized controlled trials. J. Funct. Foods.

[14] Gad-Elkareem M.A., Abdelgadir E.H., Badawy O.M., Kadri A. (2019). Potential antidiabetic effect of ethanolic and aqueous-ethanolic extracts of Ricinus communis leaves on streptozotocin-induced diabetes in rats. PeerJ.

[15] Garg P., Morris P., Fazlanie A.L., Vijayan S., Dancso B., Dastidar A.G., Plein S., Mueller C., Haaf P. (2017). Cardiac biomarkers of acute coronary syndrome: From history to high-sensitivity cardiac troponin. Intern. Emerg. Med..

[16] Hijazi Z., Oldgren J., Wallentin L., Andersson U., Connolly S.J., Yusuf S., Ezekowitz M.D., Hohnloser S.H., Reilly P.A., Vinereanu D. (2012). Response to letter regarding article, “Cardiac biomarkers are associated with an increased risk of stroke and death in patients with atrial fibrillation: A randomized evaluation of long-term anticoagulation therapy (RE-LY) substudy”. Circulation.

[17] Sandamali J.A.N., Hewawasam R.P., Jayatilaka K.A.P.W., Mudduwa L.K.B. (2021). Cinnamomum zeylanicum Blume (*Ceylon cinnamon*) bark extract attenuates doxorubicin induced cardiotoxicity in Wistar rats. Saudi Pharm. J..

[18] Xu C., Zhang T., Zhu B., Cao Z. (2020). Diagnostic role of postmortem CK-MB in cardiac death: A systematic review and meta-analysis. Forensic Sci. Med. Pathol..

[19] Moravej Aleali A., Amani R., Shahbazian H., Namjooyan F., Latifi S.M., Cheraghian B. (2019). The effect of hydroalcoholic Saffron (*Crocus sativus* L.) extract on fasting plasma glucose, HbA1c, lipid profile, liver, and renal function tests in patients with type 2 diabetes mellitus: A randomized double-blind clinical trial. Phytother. Res..

[20] Tan Y., Zhang Z., Zheng C., Wintergerst K.A., Keller B.B., Cai L. (2020). Mechanisms of diabetic cardiomyopathy and potential therapeutic strategies: Preclinical and clinical evidence. Nat. Rev. Cardiol..

[21] Ali S.S., Ahsan H., Zia M.K., Siddiqui T., Khan F.H. (2020). Understanding oxidants and antioxidants: Classical team with new players. J. Food Biochem..

[22] Zhang X., Shi E., Yang L., Fu W., Hu F., Zhou X. (2019). Gentiopicroside attenuates diabetic retinopathy by inhibiting inflammation, oxidative stress, and NF-κB activation in rat model. Eur. J. Inflamm..

[23] Hameister R., Kaur C., Dheen S.T., Lohmann C.H., Singh G. (2020). Reactive oxygen/nitrogen species (ROS/RNS) and oxidative stress in arthroplasty. J. Biomed. Mater. Res. Part B Appl. Biomater..

[24] Shi X., Xu L., Zhang J., Mo J., Zhuang P., Zheng L. (2023). Oxyresveratrol from mulberry branch extract protects HUVECs against oxidized Low-density Lipoprotein-induced oxidative injury via activation of the Nrf-2/HO-1 pathway. J. Funct. Foods.

[25] Raish M., Ahmad A., Jardan Y.A.B., Shahid M., Alkharfy K.M., Ahad A., Ansari M.A., Abdelrahman I.A., Al-Jenoobi F.I. (2022). Sinapic acid ameliorates cardiac dysfunction and cardiomyopathy by modulating NF-κB and Nrf2/HO-1 signaling pathways in streptozocin induced diabetic rats. Biomed. Pharmacother..

[26] Lind M., Hayes A., Caprnda M., Petrovic D., Rodrigo L., Kruzliak P., Zulli A. (2017). Inducible nitric oxide synthase: Good or bad?. Biomed. Pharmacother..

[27] Kitakata H., Endo J., Hashimoto S., Mizuno E., Moriyama H., Shirakawa K., Goto S., Katsumata Y., Fukuda K., Sano M. (2021). Imeglimin prevents heart failure with preserved ejection fraction by recovering the impaired unfolded protein response in mice subjected to cardiometabolic stress. Biochem. Biophys. Res. Commun..

[28] Wang X., Tan Y., Xu B., Lu L., Zhao M., Ma J., Liang H., Liu J., Yu S. (2018). GPR30 Attenuates Myocardial Fibrosis in Diabetic Ovariectomized Female Rats: Role of iNOS Signaling. DNA Cell Biol..

[29] Moradipour A., Dariushnejad H., Ahmadizadeh C., Lashgarian H.E. (2022). Dietary flavonoid carvacrol triggers the apoptosis of human breast cancer MCF-7 cells via the p53/Bax/Bcl-2 axis. Med. Oncol..

[30] Zheng Q., Wang H., Hou W., Zhang Y. (2021). Use of Anti-angiogenic Drugs Potentially Associated with an Increase on Serum AST, LDH, CK, and CK-MB Activities in Patients with Cancer: A Retrospective Study. Front. Cardiovasc. Med..

[31] Okagu I.U., Ndefo J.C., Aham E.C., Udenigwe C.C. (2021). Zanthoxylum Species: A comprehensive review of traditional uses, phytochemistry, pharmacological and nutraceutical applications. Molecules.

[32] Iftikhar A., Aslam B., Iftikhar M., Majeed W., Batool M., Zahoor B., Amna N., Gohar H., Latif I. (2020). Effect of Caesalpinia bonduc Polyphenol Extract on Alloxan-Induced Diabetic Rats in Attenuating Hyperglycemia by Upregulating Insulin Secretion and Inhibiting JNK Signaling Pathway. Oxidative Med. Cell. Longev..

[33] Dai J., Mumper R.J. (2010). Plant Phenolics: Extraction, Analysis and Their Antioxidant and Anticancer Properties. Molecules.

[34] Prakash V., Saxena S., Gupta S., Saxena A., Yadav R., Singh S. (2015). Preliminary phytochemical screening and biological activities of Adina cardifolia. J. Microb. Biochem. Technol..

[35] Ganzera M., Greifeneder V., Schwaiger S., Stuppner H. (2012). Chemical profiling of Edelweiss (*Leontopodium alpinum* Cass.) extracts by micellar electrokinetic capillary chromatography. Fitoterapia.

[36] Olegário L.S., Andrade J.K.S., Andrade G.R.S., Denadai M., Cavalcanti R.L., da Silva M.A.A.P., Narain N. (2019). Chemical characterization of four Brazilian brown propolis: An insight in tracking of its geographical location of production and quality control. Food Res. Int..

[37] Prieto P., Pineda M., Aguilar M. (1999). Spectrophotometric Quantitation of Antioxidant Capacity through the Formation of a Phosphomolybdenum Complex: Specific Application to the Determination of Vitamin E. Anal. Biochem..

[38] El-Hawary S.S., El-Hefnawy H.M., Osman S.M., El-Raey M.A., Mokhtar Ali F.A. (2021). Phenolic profiling of different Jasminum species cultivated in Egypt and their antioxidant activity. Nat. Prod. Res..

[39] Re R., Pellegrini N., Proteggente A., Pannala A., Yang M., Rice-Evans C. (1999). Antioxidant activity applying an improved ABTS radical cation decolorization assay. Free Radic. Biol. Med..

[40] No O.T. (2002). 423: Acute oral toxicity-acute toxic class method. OECD Guidelines for the Testing of Chemicals, Section 4.

[41] Alelign T., Chalchisa D., Fekadu N., Solomon D., Sisay T., Debella A., Petros B. (2020). Evaluation of acute and sub-acute toxicity of selected traditional antiurolithiatic medicinal plant extracts in Wistar albino rats. Toxicol. Rep..

[42] Barry A.J., Groseclose M.R., Castellino S. (2019). Quantification and assessment of detection capability in imaging mass spectrometry using a revised mimetic tissue model. Bioanalysis.

[43] De Leon J.A.D., Borges C.R. (2020). Evaluation of oxidative stress in biological samples using the thiobarbituric acid reactive substances assay. JoVE.

[44] Adeyi O.A., Idowu A.B., Mafiana C.F., Oluwalana A.S., Ajayi O.L., Akinloye A.O. (2012). Rat model of food-induced non-obese-type 2 diabetes mellitus: Comparative pathophysiology and histopathology. Int. J. Physiol. Pathophysiol. Pharmacol..

[45] Siddique T., Awan F.R. (2016). Effects of Reg3 Delta Bioactive Peptide on Blood Glucose Levels and Pancreatic Gene Expression in an Alloxan-Induced Mouse Model of Diabetes. Can. J. Diabetes.

[46] Abdel-Wahab N., Tayar J.H., Fa’Ak F., Sharma G., Lopez-Olivo M.A., Yousif A., Shagroni T., Al-Hawamdeh S., Rojas-Hernandez C.M., Suarez-Almazor M.E. (2020). Systematic review of observational studies reporting antiphospholipid antibodies in patients with solid tumors. Blood Adv..

[47] Ritchie R.H., Love J.E., Huynh K., Bernardo B.C., Henstridge D.C., Kiriazis H., Tham Y.K., Sapra G., Qin C., Cemerlang N. (2012). Enhanced phosphoinositide 3-kinase(p110α) activity prevents diabetes-induced cardiomyopathy and superoxide generation in a mouse model of diabetes. Diabetologia.

